# *‘In the shower crying…but we came back in the following day and did it all again’*. Distress and resilience in care home staff during the COVID-19 pandemic– A qualitative interview study

**DOI:** 10.1186/s12877-024-04804-w

**Published:** 2024-03-27

**Authors:** Zoë Cockshott, Siân Russell, Rachel Stocker, Jo Knight, Suzanne Mason, Barbara Hanratty, Nancy Preston

**Affiliations:** 1 International Observatory on End of Life Care, Faculty of Health and Medicine, Lancaster University, Bailrigg, Lancaster UK; 2 Population Health Sciences Institute, Faculty of Medical Sciences, Newcastle University, Newcastle upon Tyne, UK; 3 School of Biomedical, Nutritional and Sport Sciences, Faculty of Medical Sciences, Newcastle University, Newcastle upon Tyne, UK; 4 Lancaster Medical School, Faculty of Health and Medicine, Lancaster University, Bailrigg, Lancaster UK; 5 Sheffield Centre for Health and Related Research, School of Medicine and Population Health, University of Sheffield, Sheffield, UK

**Keywords:** Care homes, Long-term care facilities, Nursing homes, COVID-19, Staff well-being, Staff mental health, Coping, Resilience

## Abstract

**Background:**

Care homes (long-term care facilities) were profoundly impacted early in the COVID-19 pandemic, both in terms of resident mortality and restrictions for infection control. This study investigated the impact on the emotional well-being of care home staff of challenges faced at this time, and the strategies used to manage them.

**Methods:**

Semi-structured interviews conducted October 2020-June 2021 with care home staff and health service staff working with them explored the impact of the early waves of the COVID-19 pandemic (March 2020-June 2021). Interview data were analysed using reflexive thematic analysis.

**Results:**

Interview participants were 16 care home staff and 10 health service staff. Analysis generated four key themes: *1)Anxiety and distress, 2)Overwhelming workload, 3)Pulling through;* and *4)Resilience in a time of crisis.* Care home staff experienced *Anxiety and distress* due to uncertainty of what to expect; witnessing illness and deaths of residents; concerns regarding their own health, and sometimes feeling their work was under-recognised. They also experienced an *Overwhelming workload* due to infection control measures, caring for sick residents and reduction in external healthcare support. Our theme of *Pulling through* reflects the peer support and problem-solving strategies with which care home staff managed the impact of the pandemic, along with a sense of responsibility and meaning towards their work. An overarching theme of *Resilience in a time of crisis* drew on the other three themes and describes how many staff managed, maintained, and often increased their work despite the challenges of the pandemic. Participants also described increasing emotional fatigue as the pandemic continued.

**Conclusions:**

This paper builds on literature on the emotional impact of the pandemic on care home staff, also exploring ways that staff responded to this impact. These findings can help inform planning for future crises including disease outbreaks, and raise important questions for further work to develop pandemic preparedness in care homes and beyond. They also raise wider questions about the current cultural status of care work, which may have exposed care home staff to greater risk of distress, and which contrasts with the professionalism and responsibility shown by staff in response to pandemic challenges.

## Background

### COVID-19 in care homes

Care homes (or long-term care facilities) worldwide faced multiple and significant challenges during the early waves of the COVID-19 pandemic [1–6]. Due to their communal occupancy and the physical vulnerability of residents, care homes were heavily impacted in terms of illness and mortality. Care home residents’ deaths accounted for 47% of COVID-19 deaths in England and Wales during the first UK wave of the pandemic in Spring/Summer 2020 [7], and 35.2% of all COVID-19 deaths in the first year of the pandemic from March 2020-April 2021 [7], with similar rates of 30–41% reported across this period internationally [6, 8].

During the early stages of the COVID-19 pandemic, care home staff were faced with managing high levels of illness and death amongst residents, as well as multiple demands of infection control tasks [9]. These arose in a context of rapidly and frequently changing government regulations and policy, which often compounded difficulties in access to support and resources, [4, 10] and which led to the discharge of patients with COVID-19 from hospitals into care homes [4, 39]. These challenges were managed alongside significant risk of contracting COVID-19 and its potential impact on their own health and that of their families.

A growing body of literature indicates that the COVID-19 pandemic had a significantly negative impact on the emotional as well as physical well-being of staff across health and social care sectors internationally. Studies of healthcare staff during the period have found increased levels of anxiety, depression and burnout [11–15] and moral distress [16, 17]; defined as ‘*the experience of being seriously compromised as a moral agent in practicing in accordance with accepted professional values and standards*’ [18] pp 59.

Other studies have focused specifically on the impact of the pandemic on the well-being of care home staff. During the first waves of the pandemic, attention was drawn to the risk factors for emotional impact in this group of workers, with some authors referencing pre-pandemic challenges already faced by the sector [2, 10, 19]. Pandemic aside, the nature of care work presents a complex and challenged picture. The sector has a historically low-paid, often under-recognised workforce providing care for residents with multiple and complex health and social care needs [1, 10]. The demands of such work can lead to high levels of burnout and staff turnover [20–22]. Concerns regarding the impact of the pandemic on this workforce have been borne out as recent studies confirm experiences of moral distress/injury [23, 24], increased workload, increased levels of burnout and staff sickness, and a deterioration in mental well-being amongst care home and nursing home staff during the pandemic [23–26]. Birt et al. (2023) [23] focused on registered nursing staff in care homes and, as well as identifying the impacts of the pandemic on this staff group, also identified factors they used to mitigate these impacts. These included solidarity and peer support, along with an increased sense of responsibility and of their own skills and ability to provide care in a crisis with limited external support.

Some authors have suggested that resilience might mediate the impact of the pandemic on the well-being of care home staff [4, 27]. The concept of emotional resilience in the field of health and social care has gained increasing attention over the last two decades [28–30], with suggestions that resilience may lessen the impact of stress and burnout in healthcare staff [30–32]. Whilst definitions vary, most reflect resilience as *‘the ability to bounce back and carry on with life after adversity or trauma*’, with key characteristics of *rebounding and carrying on; a sense of self; determination; and a prosocial attitude* (positive relationships with others) [33]. In a scoping review, Johnston et al. (2021) [27] suggested four key factors which might enhance and support care home staff work-related wellbeing and resilience, in the face of the challenges of the pandemic: *Culture of care* (within the care home); *Content of work; Connectedness with colleagues* (peer /social support), and *Characteristics of leaders in care homes.* Marshall et al. (2020), [4] following interviews with care home managers, identified examples of organisational and collaborative resilience and resourcefulness in the way that care homes, as organisations, dealt with the early challenges of the pandemic, both internally and in collaboration with other homes and partners.

## Methods

### Study aims

The aim of this study was to explore experiences of COVID-19 for care home staff in care homes in the early stages of the pandemic (March 2020-June 2021), as reported from interviews with care home staff working during the period, and health service staff working closely with them. The findings reported here formed part of a dual-focus interview study involving care homes 1 in an area of the North of England. The other part of the study evaluated the roll-out and use of a Digital Care Home Referral and Monitoring Service (Digital Care Home Service) in the context of the COVID-19 pandemic [34]. Specifically, this part of the project aimed to:

- Build upon the findings of other authors [23–26], exploring the emotional impact of the early stages of the COVID-19 pandemic on care home staff, from the perspective of care home staff and of health service staff working closely with them;
- Explore how care home staff managed this impact, from the perspective of care home staff and that of healthcare staff working closely with them, with a particular focus on the use of personal coping strategies and resilience.

### Participant selection and recruitment

Participants were care home staff and health service staff working closely with them, including community nursing staff (clinical staff) and the Digital Care Home Service Team (administration and training staff). They were recruited from the area covered by the Digital Care Home Service.

#### Sampling

Initially, purposive sampling was used for the recruitment of care home staff and health service staff to include a broad range of participants in terms of care home size and type (nursing/residential homes), and staff roles. Interviews took place October 2020-June 2021, during the UK’s second wave of the COVID-19 pandemic so recruitment was challenging, and sampling was, by necessity, often pragmatic and opportunistic. Care home staff were recruited to the study irrespective of current health status, or length of service in the care home. Study exclusion criterion was inability to give informed consent (which did not arise in staff recruitment).

#### Recruitment

Care home managers and health service staff received initial contact from the Digital Care Home Service manager, who sent out two recruitment emails in Autumn 2020 and Spring 2021. For care home staff recruitment, emails advertising the study were sent to all care homes in the area using the Digital Care Home Service (approximately 100) inviting them to participate and giving contact details for the research team. Initial contact was made with the person in the care home who had responded to this recruitment call, and this key contact (usually the Care Home Manager/ Deputy Manager) was asked to cascade the information and invitation to their staff. For health service staff, the Digital Care Home team made direct introductions between the research team and individual members of staff. In both cases, individuals agreeing to take part in the study were contacted directly by one of three researchers and invited to participate in an interview. Informed consent was obtained either electronically, or verbally and audio-recorded in line with Health Research Authority (HRA) guidance.

### Data collection

Semi-structured, qualitative interviews were conducted either by online video-call or telephone. The topic guide for the full interview included questions about the impact of COVID-19 on the care home, staff and residents, (including considering the period from the first wave of the pandemic to the time of interview, during the second wave), as well as questions regarding use of the Digital Care Home Service. The questions regarding the impact of COVID-19 were open-ended and designed to explore the impact of visiting restrictions and infection control requirements, of COVID-19 cases and deaths, and changes in access to health care services. Interviews were conducted by one of the three researchers (ZC, SR, RS), and were either one-to-one, or in small groups (two/three staff), depending on participant preferences. Interviews were audio-recorded, transcribed verbatim using a secure service, and anonymised.

### Data analysis

A reflexive thematic analysis approach was used, drawing on the guidelines of Braun and Clark’s (2006, 2019) [35, 36] six-phase framework; *familiarisation with data, generating initial codes; searching for themes; reviewing themes; defining themes and writing up analysis*. The researchers (SR, RS and ZC) each read a sample of early interviews *(familiarisation)*, and then collaboratively *generated initial codes* based on inductive coding from this familiarisation, and from a set of a priori codes based on similar previous work by members of the research team (RS, SR, BH). Codes were regularly reviewed and revised iteratively following analysis of further transcripts, and through periodic meetings between the researchers and the wider study team (NP, BH). As data collection and analysis continued, the research team compared codes within and between transcripts to *search for themes*, and then continued the reflexive process to *review* and *define themes*. Analysis was then further refined by consideration of key research questions for the purposes of reporting and dissemination (*writing up analysis*). For the purposes of this paper the focus was on themes relating to the impact of the pandemic on the emotional well-being of care home staff and its management.

### Public involvement

A public involvement group was established which included members of the public with an interest in care homes and social care, through experience as a carer or having worked in the sector. This group was consulted at the initial stages of design of the study, and during data analysis (June 2021). Anonymised sections of transcripts were shared with the group to elicit ideas on important questions to address from the data which helped guide the focus of analyses.

## Results

A total of 20 interviews were conducted with 26 participants: sixteen care home staff from eight care homes, and ten health service staff. Details of the roles of individual participants are shown in Table 1. Details of the eight participating care homes are shown in Table 2. Sixteen interviews were one-to-one, four were in pairs or small groups (participants’ choice). Seventeen were conducted online, one by phone and two were a combination of online and phone. Interview duration was between 31 and 68 min within a mean length of 50 min.

**Table 1:** Participants by setting and role

Setting	Role (& abbreviation for participant IDs for interview quotes)	Participants
**Care Homes**	Care Home Managers (CHM)	4
	Deputy Managers (CHDM)	3
	Senior Carer / Carer (SC/JC)	9
	**Care Home Sub-total**	**16**
**Health Service**	Community nursing staff (CN)	6
	Digital Care Home Service Team (DCHS)	4
	**Health Service staff Sub-total**	**10**
	**Grand Total**	**26**

**Table 2:** Summary of Care Homes recruited by provider type, care type and size

Care Home	Participants	Type of Care Home Provider	Care provided	Care Home size (no. of beds)
1	2 x Senior Carers 1 x Carer	Chain	Residential	~ 60
2	1 x Deputy manager 1 x Senior Carer	Chain	Residential & Nursing	~ 50
3	1 x Deputy manager 1 x Senior Carer	Chain	Residential & Nursing	~ 60
4	1 x Care Home Manager	Independent	Residential	~ 25
5	1 x Care Home Manager 1 x Deputy manager 1 x Senior Carer	Chain	Residential	~ 50
6	1 x Care Home Manager	Chain	Residential	~ 70
7	1 x Care Home Manager 2 x Senior Carer	Chain	Residential	~ 70
8	1 x Senior Carer	Independent	Residential & Nursing	~ 20

All Care Homes had Care Quality Commision rating *‘Good’*

All Care Home staff taking part in interviews had been in their current position since before the start of the pandemic in March 2020. (Duration in current role: Range = 2–25 years, mean = 8.5 years)

### Qualitative findings

Thematic analysis generated four key themes relating to the emotional impact of the pandemic and how staff managed its challenges; (1) *Anxiety and distress*, (2) O*verwhelming workload, (3) Pulling through;* and (4) an overarching theme of *Resilience in a time of crisis.*

In interviews, staff described how the arrival of COVID-19 profoundly impacted many aspects of their work and personal life and was experienced as stressful. Most reflected that it had been an exceptionally difficult time due to anxiety and distress regarding the disease itself and witnessing its impact on residents’ physical health and social connections. They also experienced significantly increased workloads due to infection control tasks, caring for severely ill residents and high levels of sickness absence, and sometimes felt abandoned and under-valued by health services, policy makers and the public. Thus, the emotional impact of the COVID-19 pandemic on staff was characterised by two broad areas, our first two themes of Anxiety *and distress*, and *Overwhelming workload.* The two were often closely interlinked and combined to create a challenging working experience.

### Anxiety and distress

The anxiety and distress experienced by care home staff during the early waves of the pandemic was multifaceted and arose from a number of factors. Many staff reported a high level of fear and uncertainty especially early on, when there were frequent reports of COVID-19 illness and deaths in other countries. Staff had seen news from overseas where care homes had high rates of COVID-19 deaths, and they awaited the arrival of COVID-19 in their care homes with a sense of dread. This was compounded by being asked to make preparations beyond any previous experience or expectations.

*‘Just before lockdown I think, we had a nurse came to the home and said to us, ‘Right, you need to be prepared to hold bodies in the care home. Do you have any cold bedrooms where you can hold bodies?’….And I think that kind of hit us like, ‘wow.’ We were thinking, ‘God I’m not sure what’s going to happen. How are we going to cope with this…?’ kind of thing.*’ CHM1.

When the anticipation of COVID-19 was met with the reality of an outbreak, staff were distressed by the illness and death of residents and shocked at both how quickly the disease spread through the care home, and how quickly it could impact on individual residents. Staff frequently used terms like ‘horrific’, and ‘awful’ when describing these events.

*‘I mean it was awful because we knew it was going to eventually happen… because obviously other homes in [Town] had had it, and we’re like, ‘It’s coming’, it’s just a case of trying to keep it off as long as possible. And unfortunately, it was horrific for us because obviously a lot of our people are very vulnerable… we unfortunately lost, I think it was 11 residents we lost, and one member of staff to COVID over a space of about three weeks.’* JC1.

In addition to concerns about residents, care home staff were anxious about risk to themselves and colleagues of exposure to COVID-19, and about taking infection home to family members.

*‘I lived with my mum at the time and my mum is quite vulnerable. She was told to shield… it was just really hard for everyone; not seeing the family, having to make sure my mum was okay and look after all the residents that were poorly…. It was just stressful, yeah.’* SC7.

#### Feeling abandoned, undervalued, and criticised

Accounts suggested that care homes sometimes felt ‘forgotten’ and left to fend for themselves during the early challenging months of the pandemic, which added to their distress. Health service staff noted that there had been a sense of abandonment in care homes at the start of the pandemic when their usual nursing and medical support switched to more remote delivery of care, which some care home staff perceived as a withdrawal of healthcare support. Some care home staff felt under-valued in comparison to acute health services, both in terms of provision of resources such as Personal Protective Equipment (PPE), and in terms of recognition of their role. This feeling was compounded by the sometimes critical judgement of the press.

*‘Some of the homes saw the district nurses and… the community matrons disappear… so, there was some, I don’t want to use ‘ill feeling’ but there was some sort of sense of abandonment.’* DCHS2.

*‘What they’re saying [media coverage], quite often you’re like, ‘No, that’s not actually what happens’…And I don’t think that care homes were necessarily shown in the best light’* SC3.

#### Moral distress

Much of the distress of care home staff arose from a sense of personal and professional responsibility for protecting residents from COVID-19 and its impact. Staff often expressed feelings of guilt and responsibility when they had an outbreak in their care home.

*‘They [families of residents with COVID-19] were so grateful even though it was a COVID situation, and we felt really guilty about it, kind of thing.’* DM1.

This sense of responsibility was a recurring theme in interviews and may have contributed to moral distress, when staff felt unable to meet their professional and personal values for care due to constraints of COVID-19. Consistent with the notion of moral distress, care home staff often expressed concern about the impact of the pandemic on the type of care that they were able to provide to residents, particularly in terms of providing them with social interaction and stimulation. Staff were concerned about the impact of restrictions on residents’ quality of life in terms of family visits, particularly towards the end of life;

*‘Obviously I’ve done end of life care many times in the past but I think it was more awful knowing that this didn’t need to be happening, this wasn’t standard end-of-life care we would be doing.’ JC*.

*‘When you’ve got a relative in the room and you’re like, ‘I’m sorry your time is up. You’ve got to go.’ It’s just an awful position to be in because who are we to say they can’t say their goodbyes and for how long. That’s the bit that I find difficult because I just think it’s awful. It really is.’ SC6*.

To summarise, care home staff experienced considerable anxiety, and emotional and moral distress at the beginning of the pandemic, in the face of an unprecedented health emergency. They often felt isolated from wider healthcare provision and were caring for highly vulnerable residents, who often became very sick very rapidly.

### Overwhelming workload

Care home staff frequently described having very high workload demands in the early months of the pandemic. Workload was increased for many reasons, including the implementation of infection controls, caring for severely ill residents, a reduction in external support and staff sickness absence.

#### Infection control tasks

Throughout the first and second UK waves of the pandemic, government guidelines for infection control were changing frequently and rapidly, meaning that extra work was needed to interpret and accommodate changes. These requirements often felt like they added further to the demands on staff when they already felt extremely busy and overstretched. Staff often described a sense of information overload.

*‘I think to start off with, the amount of information that was coming through to us was just unbelievable. We had local authority, department of health and social care. I get direct emails from them.… CCG, local authority, department of health and social care, NICE guidelines. CQC sent us things out. It was coming from all directions! Yeah we’ve had it from government level as well.’* CHM1.

Care home staff were required to make physical changes to the layout of homes to allow for social distancing and infection control and to implement and enforce visiting and testing rules. Some staff expressed frustration at having COVID-19 infection control guidelines from central Government, noting that infection control was an intrinsic part of care home work pre-pandemic. They felt the imposition of these centralised guidelines reflected a long-standing misunderstanding of the day-to-day work and skills of care home staff.

*‘We were doing everything that you need to do beforehand.…We were doing it anyway.… if you had stringent policies in place beforehand I don’t think it needs to have the word COVID on just to prove that you’re doing it.’* CHDM2.

#### Managing COVID-19 outbreaks and resident illness

Care home staff described the times when they had a COVID-19 outbreak as particularly demanding in terms of workload, as well as being distressing. Despite infection control measures, COVID-19 often spread rapidly between residents, meaning that staff needed to care for several seriously ill residents and their families within a short period. At times of a COVID-19 outbreak, infection control was even more challenging, especially when supporting residents with dementia. Some of these residents needed frequent reminders about social distancing and changes in layout of the home. It was difficult restricting them to one space when they were supposed to be isolating. Residents often required one-to-one attention, and staff described having to follow residents with dementia around the home ensuring that they were not in contact with others.

*‘I mean COVID in general has just added to our workload– the whole COVID situation, but mainly on the dementia unit… When we did have the outbreak,…they were coming out of the rooms when they had COVID, we had to continuously walk behind them to put them back into the room, but with the cleaning products as well so no one else touched that area. It was just all systems go really.’* SC6.

#### Managing contacts with families

Due to restrictions on visiting, care home staff had to find additional and innovative ways for residents to communicate with their families and the wider community, including the rapid implementation of online communications with family via video calls and social media groups. Staff were also dealing with a number of concerns and queries from residents’ families, especially at times of changes in government guidelines, all adding to workload pressure as well as moral distress. They often had to manage expectations of families who were upset or angry that they were unable to visit or having to wait for COVID-19 test results before doing so. Tensions over family visiting also sometimes provoked negative reports in the media which could be an added pressure for care home staff.

*‘It has been really difficult, but like we say the carers have gone above and beyond in regards to trying to keep that communication and trying to keep the video calls etc. and have the window visits. Or if there’s activities going on we’ll take pictures, we’ll send them to the family just keeping that involvement…, they know what’s going on in the home. They can’t come in so yeah, the residents, it’s upsetting for them.’ SC*7.

#### Fewer staff for more tasks

An additional factor contributing to the workload of care home staff, was that they increasingly had to perform nursing tasks normally undertaken by external healthcare staff who had reduced care home visiting early in the pandemic. Care home staff were also dealing with the risk and reality of becoming unwell with COVID-19 themselves and staff sickness absences increased the workload and pressure on colleagues.

*‘I felt like absolute rubbish [returning to work after having COVID-19], but it was like you needed to be back in and you needed to be doing stuff. You needed to be in there and mucking in and stuff and there wasn’t pressure for us to come back. It was my own personal like, ‘I need to be back’.’* SC3.

*‘The workload just tripled if that was even possible. Yeah, really overwhelming for everybody… It was hard.’* SC7.

In summary, staff experienced exceptional increases in workload during the early months of the pandemic due to infection control and social distancing requirements, caring for seriously ill residents, and covering for staff sickness absence and reduced access to external healthcare and other sources of support.

### Pulling through

Staff described a number of factors which they felt helped them to manage the emotional impact of the early months of the pandemic. Care home staff did receive and value support from management and the local community, but working together as a team, problem-solving, and sense of duty and responsibility appeared to be the key factors in ‘pulling through.’

#### Pulling together as a team

Many participants suggested that peer support from colleagues was central to coping with the demands of the pandemic for care home staff, with colleagues supporting one another inside and outside of work. Several care home staff described coming together as a team, almost as family, in their response to the challenges. A sense of feeling closer and stronger as a team was evident from interviews. Managers also noted this team ethos amongst their staff and saw the process of ‘pulling together’ as an important factor in getting through challenging times.

*‘Everyone really pulled together as a team so that was nice to see. We supported each other in and outside of work as well… It has been hard. Some people have struggled more than others. We did have some that did [leave] for health reasons, but everyone that has worked right through has pulled together and like I say, become closer, if anything, because of it all.’* SC7.

*‘We’ve all been mucking together and I think there’s a mutual respect in terms of that and they really have. They’ve worked really well and they’ve just gotten stuck in and just got on with it. Done really well.’* CHDM2.

#### Problem-solving

Staff often also used personal and problem-solving strategies when responding to some of the distressing challenges posed by the pandemic. The sense of ‘getting on with it’ suggested in the quote above was reflected in several comments from care home staff. Many described being motivated to continue with their work out of a sense of responsibility, commitment, and meaningfulness of their role also suggesting that they perceived their work as important and valuable. This sense of value and of needing to ‘get on’ with the job and to look after the people in their care may have contributed to meaning-making, or *meaning-based coping*.

*‘I think we just went into like an overdrive mode. We had to, sort of… I felt that I had to put my feelings aside for the residents in here.’ SC6*.

Care home staff also often used problem-solving approaches to resolve some of the distressing impacts of the pandemic on residents and themselves. They worked proactively to overcome various challenges including restrictions on visitors and others coming into the home. Substantial efforts were made to help residents communicate with their families online, by phone, or in ‘visiting pods’, and to organise online social events. Staff also came in on their days off to perform tasks such as hairdressing that would normally be performed by someone external to the home. They were also often proactive in approaches to infection control suggesting a much higher level of agency and professionalism than simply passively following government infection control guidelines. Some described infection control procedures that they were putting in place *prior* to central government advice.

‘*Like infection control would ring and go, ‘Oh we’ve got this information now. You need to put this in place.’ I’m like, ‘We already did that three weeks ago…’ I think we were really proactive in changing stuff before it happened’* CHDM1.

In summary, staff used a range of strategies to manage the emotional impact of the pandemic, drawing on a combination of a sense of responsibility for their role, practical problem-solving and both giving and receiving peer support.

### Resilience in a time of crisis

Resilience in a time of crisis is considered our over-arching theme since it is an observation which draws on our other three themes. Resilience, by definition, relies on both the experience of adversity (in this case the COVID-19 pandemic resulting in anxiety, distress and overwhelming workload) and managing *despite* that adversity (pulling through). Therefore, this theme was generated following an analytic overview of the other three themes, and also encompasses responses which specifically suggest managing *despite* the challenge.

The sense of commitment and responsibility to ‘get on with it’, along with proactive problem solving, provide a strong indication of resilience amongst care home staff in this study. Mindful of definitions of resilience as a process of perseverance and ‘bouncing back in the face of adversity’, this process was reflected in staff interviews;

*‘I mean to be fair to myself and everybody else, never let it show though. You know we’d all go home and we’d all be like, ‘Oh god I’ve been in the shower crying,’ and you know, it affected us when we got home, but we came back in the following day and we did it all again and it was just part of life.’* SC6.

*‘You’d go into some different mode working through the early days of it.’ SC7*.

Care home managers recognised the pressures that staff had been under and reflected that some staff had left due to these pressures, but also noted the resilience of many staff, and their ability to continue work in the face of challenging circumstances. This was also reflected from the external perspective of health service staff who described the way that care home staff had responded to the pandemic, and how care home staff had gained skills.

*‘They’ve been fab. They’ve been very keen to manage their own patients… but they’re not sort of shouting for help when actually they can do. I think they’ve learned that they can do a lot more and we just need to sort of appreciate that really and it’s definitely an upskilling for them.’ CN4*.

Health service staff did however note that this response was not universal amongst care homes they visited, and looking to the future, some expressed a note of caution that the repercussions of the pandemic in care homes may be felt for some time to come. Similarly, care home managers noted that the pandemic had been tiring, suggesting that there might only be so long that such a response could be maintained.

*‘I think they’ve coped really well but we won’t know the fallout of all of this for another 12 months.’* CN1.

That care home staff showed considerable resilience in the early months of the pandemic is evidenced by their accounts of the overwhelming workload, anxiety and distress they experienced, and their adaptive and proactive responses to its impact. It is also important to note that care home staff, their managers, and those working closely with them recognised that they were meeting the demands of the pandemic, at many times with resourcefulness, skills and commitment that they had not previously known they had.

## Discussion

The findings reported here indicate that the early waves of the COVID-19 pandemic were experienced as extremely challenging for care home staff. Whilst care home staff in this study generally reported feeling well-supported by management, their accounts more strikingly indicate that they responded to these challenges through team-work and peer support, proactive problem-solving and a sense of responsibility to their residents suggesting considerable resilience. These findings are illustrated not only from accounts of care home staff themselves, but from observations of health service staff working with them.

The findings of the emotional impact of the pandemic reported here reflect those of others describing the impact on healthcare [11–15] and care home [19, 23–26] staff. The accounts of high levels of anxiety and distress, and greatly increased workload in the early months of the pandemic have resonance with the ‘Guilt, tears and burnout’ reflected in the title of Giebel et al’s (2022) [25] paper. Our findings also indicate that staff experienced moral distress [18] when they felt that their ability to fulfil their care role was compromised. This was particularly pronounced when care home staff needed to restrict relatives’ access to residents and echoes the findings of other authors of moral distress for care home staff [23, 24] and palliative care staff [16, 17] during the pandemic. The findings here build on the work of other authors investigating the emotional impact of the pandemic on care home staff, but from the dual perspective of care home and health service staff, and also reflect on how this group of staff managed these challenges. They highlight the importance of peer support and a sense of duty and responsibility in managing the impact of the pandemic, reflecting the findings of Birt et al. (2023) [23] amongst nursing staff in UK care homes. Here we are able to build on this work, suggesting that these types of responses were prevalent across a wide range of care home staff, most of whom were not registered nurses.

Whilst some of the stressors identified by participants in this study reflected those common to a range of healthcare staff, others appeared to be more specific to the nature of the work of care homes, and the cultural climate in which they are placed. These include the tensions of maintaining constructive relationships with residents’ families despite COVID-19 restrictions, and often feeling left to care for the complex health needs of residents with limited resources or support from outside agencies. Care home staff sometimes felt that their needs, efforts and achievements were undervalued compared to those of the health service, and that care homes were at times strongly criticised in the media.

### Resilience, responsibility and going above and beyond

The findings that many care home staff met the challenges of COVID-19 with individual and group resilience, and innovative and proactive solutions, has parallels with the findings of *organisational* resilience across care homes in the face of COVID-19 reported by Marshall et al. 2020 [4]. Regarding the ways care home staff managed the emotional impact of the pandemic, our findings reflect characteristics of resilience [33], namely the processes of *bouncing back* from significant sources of stress or trauma and *rebounding and carrying on*, through *a sense of self, determination and a prosocial attitude*. A sense of self and determination *(or ‘getting on with it’) we*re particularly pronounced in the expressions of problem-solving and meaning-making strategies. Interviews suggest that staff responded to problems proactively and with a sense of professional responsibility. Powell et al. (2020) [30] suggest that a sense of ‘making a difference’ and ‘creating a meaningful narrative’, contributed to resilience in palliative care nurses (pre-pandemic), and this sense of meaning-making was also reflected in our findings. Staff reflected on the responsibility to fulfil what they saw as an important and valuable role and demonstrated determination in doing so. This also has resonances with the concept of *meaning-based coping* [37] which may be used in adverse circumstances where other coping strategies are not effective or appropriate. A pro-social attitude was evidenced in numerous reflections on the importance of peer support and working as a team. Indeed, our data suggest that far from being independent attributes of resilience, social support and a sense of self and determination are intrinsically interlinked; that is, that a combination of meaning-making and team support may be particularly successful in helping to achieve resilience.

Johnston et al.’s (2021) [27] four key factors for the well-being and resilience of care home staff also provide interesting comparisons with our findings. Our interviews suggested the importance of peer and organisational support in staff coping with the emotional impact of the pandemic echoing Johnston et al.’s *Connectedness with colleagues*, and *Characteristics of leaders.* However, the roles of the dimensions of *Culture of care* and *Content of work* identified by Johnston et al. (2021) [27] appear more complex. Our data suggest that the cultural climate for care homes in the UK, and the content and nature of their work during the pandemic, presented considerable challenges, but many care home staff remained resilient despite this. This raises the possibility that, in the short term at least, strength of peer and organisational support as well as personal determination is sufficient to support well-being and resilience. It is beyond the scope of this paper to examine the mechanisms for this, but one explanation is that a sense of purpose and group cohesion created a sense of satisfaction with the culture and content of their work.

The concept of resilience has attracted criticism for its over-reliance on individual rather than structural or governmental responsibility for responses to crises [38] and there are broader ethical questions about reliance on individual resilience of staff to manage crises in a sector already under pressure. It is unclear what the longer-term costs will be in terms of burnout and post-traumatic stress disorder for staff managing such pressures with limited external support. Whilst our study did not identify the extent of burnout within the sector, accounts of some staff needing to leave their work due to the demands of the pandemic suggests that resilience was by no means a universal experience. It remains to be seen whether the challenges of cultural climate and the nature of care work, especially during the pandemic, will have a lasting impact on the retention of this workforce. To date, the bulk of the media, public attention and public inquiries have focussed on the undeniably devastating costs of COVID-19 deaths and of stringent visiting restrictions on care home residents and families. It is hoped that future research, along with forthcoming public inquiries focusing on COVID-19’s impact on the care sector, will also consider the substantial costs to those working in the sector.

### Limitations

It is possible that our sample was made up of care homes and staff who were able to manage and overcome the demands of the pandemic more comfortably than others. For example, those care homes with time and staff resources to participate in a research study may have been under less pressure from the pandemic. In addition, study recruitment within care homes was generally via care home managers, who might have selected more experienced staff to participate. Our sample was weighted towards senior care staff who may have had more skills and confidence to meet the demands of the pandemic with resilience.

We were unable to interview those care home staff reported to have left their roles due to the pressure of the COVID-19 pandemic, which may have provided insights into what made it more difficult to manage the impact of the pandemic. All participants took part in one interview at a single time-point, so we were unable to track over time how responses to the pandemic developed or were maintained, and the impact that this had on the broader working lives of care home staff as the crisis subsided. Some participants were interviewed in Spring 2021, when their recall of the events of Spring 2020 may have been fading.

Despite the challenges of the continuing pandemic, we were able to recruit 16 care home staff and managers from eight care homes to this study in a short time frame, and these findings provide rich and detailed insights into the emotional impact of COVID-19 on a group of care home staff and into a range of ways in which this impact was managed.

### Future research and implications

Future research might examine the longer-term impact for care home staff in the aftermath of the pandemic, for example whether the apparent resilience of care home staff persisted, or whether there was, as some staff suggested, a growing sense of fatigue, and burnout. It might examine, as the crisis of the pandemic subsides, whether the experience of working through it had a long-term impact on the way that staff see their work in terms of their skills, problem-solving and autonomy, the way that they are perceived by the public, and what this reveals about the nature and perceived value of care work in the longer-term. Future work might also explore the experiences of those staff who left their roles due to the stresses of the pandemic, to get a fuller picture of the impact on care home staff, but also to find out whether these staff later returned to care work. Peer support and a sense of meaning and value in their work appear to be important to care home staff in managing the demands of the role, and this has wider implications for structural and local interventions to support well-being and retention of this group of staff. However, many of the sources of stress and distress for care home staff during early waves of the COVID-19 pandemic arose from structural factors, such as frequently changing policy on issues such as infection control, reduced support from outside agencies and hospital discharge to care homes. Future pandemic planning would benefit from a coordinated and integrated approach recognising the impact of such factors and addressing the needs of the care sector as a priority.

## Conclusion

Our findings indicate that care home staff found the experience of working through the pandemic emotionally demanding and distressing. In many cases, their ability to respond to these challenges through mutual support, meaning-based coping and innovative solutions suggests a resilience and professional responsibility which is inconsistent with traditional attitudes regarding the status and value of care work.

Both meaning-based coping and resilience are, by definition, strategies for managing adversity. That these strategies were demonstrated by care home staff in this study speaks to their tenacity and professionalism. However, it is unlikely to be tenable or acceptable for staff to manage adversity indefinitely, in the absence of enhanced investment and recognition for the sector.

## Data Availability

The datasets generated and/or analysed during the current study are not publicly available due to potentially identifiable confidential details from interviews, but are available from the corresponding author on reasonable request.

## Footnotes

1 In the UK, the term *care home* is generally used to refer to residential long-term care facilities. For the purposes of this paper, the generic term ‘care home’ is used for both care homes with nursing (nursing homes) which offer 24 h onsite registered nursing staff; and those without nursing (residential homes) which offer personal care, such as help with washing and dressing, but relying on external health service providers for residents’ healthcare needs.

## Abbreviations

CHM: Care Home Manager
CHDM: Care Home Deputy Manager
CN: Community Nurse
DCHS: Digital Care Home service
HRA: Health Research Authority
JC: Junior Carer
SC: Senior Carer
